# A case of acanthosis nigricans in a HIV-infected patient

**DOI:** 10.1186/s12879-020-05089-1

**Published:** 2020-05-20

**Authors:** Alessandra Iacovelli, Ivano Mezzaroma, Marcello Di Paolo, Giuseppe Soda, Ludovica De Vincentiis, Paolo Palange

**Affiliations:** 1 Department of Public Health and Infectious Diseases, “Sapienza” University of Rome, Rome, Italy; 2 Department of Translational and Precision Medicine, “Sapienza” University of Rome, Rome, Italy; 3 Department of Molecular Medicine, “Sapienza” University of Rome, Rome, Italy

**Keywords:** Acanthosis nigricans, HIV-1 infection, Antiretroviral therapy, Metabolic syndrome, Case report

## Abstract

**Background:**

To date, very little information is available concerning the relationship between *acanthosis nigricans* (AN) and infection with human immunodeficiency virus type 1 (HIV-1).

**Case presentation:**

Herein, we report the case of a middle-aged man admitted for fever and progressively worsening dyspnea in the context of an opportunistic pneumonia and firstly diagnosed with acquired immunodeficiency syndrome (AIDS). At the time of diagnosis, physical examination revealed the presence of a palpable, hyperpigmented skin lesion on the left areola with surface desquamation and velvety texture consistent with AN. Of note, the most common primary etiologies related to AN were excluded and the complete regression of the skin lesion was observed once antiretroviral therapy was started.

**Conclusion:**

This is the second report of AN found in patients with AIDS and apparently responsive to prolonged antiretroviral treatment. Possible explanations of this association are still not completely understood, probably related to virus-induced changes in lipid metabolism. Our experience suggests that HIV testing should always be considered in the setting of apparently idiopathic AN.

## Background

*Acanthosis nigricans* (AN) is characterised by a darkly pigmented skin lesion, usually localised in intertriginous areas, usually palpable with a velvety texture. Histopathological features of AN typically include epidermal and dermal hyperplasia with orthokeratotic hyperkeratosis, papillomatosis of the *stratum spinosum* and basal layer hyperpigmentation [1]. AN can be associated with several conditions, especially metabolic syndrome, insulin resistance, endocrinopathies, malignancies, and some medications [1, 2]. Interestingly, treatment is not clear, but if secondary to malignancy AN disappears with tumor eradication [1].

Findings of human immunodeficiency virus (HIV)-associated AN are anecdotal and only poor information is available from literature regarding this issue. Maltez et al. [3] firstly reported a case of a patient with AIDS who presented with three opportunistic infections and concomitant AN. In that case, AN disappeared after starting antiretroviral therapy (ART). Similarly, we here report a case of AN in the setting of a newly-diagnosed AIDS, which successfully regressed after a prolonged course with raltegravir and tenofovir/emtricitabine combination therapy.

## Case report

**Fig. 1:**
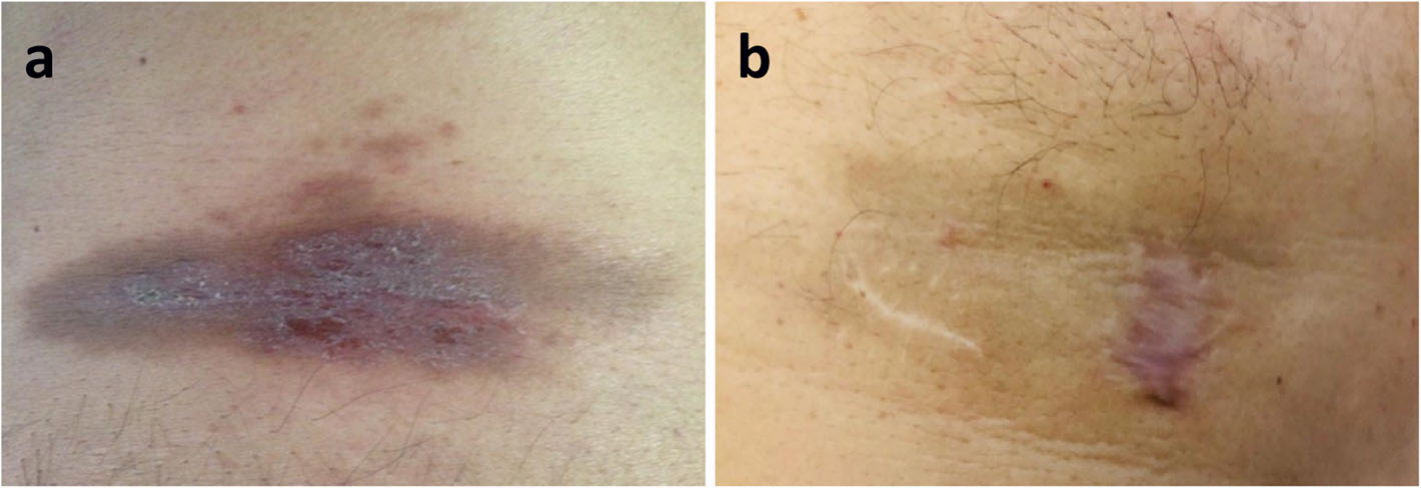
Skin lesion at the time of patient’s admission (**a**) and after one year of ART treatment (**b**). See text for further explanation

A 51-year-old man was admitted to our General Medicine Division complaining of intermittent fever (up to 40 °C) and progressively worsening dyspnea associated with fatigue and weight loss. His medical history was positive for arterial hypertension on treatment with olmesartan and hydrochlorothiazide; neither further medical disorders nor other medications were reported. On admission, physical examination revealed diffused bilateral crackles at chest auscultation and the presence of a palpable, hyperpigmented skin lesion on the left areola with surface desquamation and velvety texture (Fig. 1a). The patient had no known previous dermatosis. Oropharyngeal candidiasis was also present. No further abnormalities were found at physical examination.

Blood count revealed normocytic anemia (hemoglobin 9 g/dL) and lymphopenia (total lymphocytes 380/μL). Arterial blood gases analysis showed moderate hypoxemia (pO_2_ 71 mmHg) and hypocapnia (pCO_2_ 30 mmHg). Chest X-ray revealed the presence of multiple parenchymal infiltrates, while cysts of *Pneumocystis jirovecii* were detected in bronchoalveolar lavage samples. Diagnosis of *P. jirovecii* pneumonia was made and the patient was started on combination treatment with trimethoprim/sulfamethoxazole (160/800 mg, 2 tablets per os q8h) and fluconazole (200 mg per os q24h).

Considering the opportunistic nature of *P. jirovecii* pulmonary disease, HIV-1 infection was suspected. Serology was positive for HIV-1 antibodies at both ELISA and western blot confirmatory testing; plasma HIV-1 RNA levels revealed high viral load (325,000 copies/mL). CD4+ T cell count showed a profound immunosuppression (37 cells/μL). HIV-1 genotypic drug resistance test was then performed, showing the presence of a wild-type virus (CRF12_BF). HLA-B*5701 tested negative. Cytomegalovirus (CMV) viremia was also detected (1412 copies/mL). Therefore, diagnosis of acquired immunodeficiency syndrome (AIDS) was established and the patient was subsequently started on antiretroviral therapy (ART) with raltegravir 400 mg per os q12h and tenofovir/emtricitabine fixed-dose combination per os q24h.

**Fig. 2:**
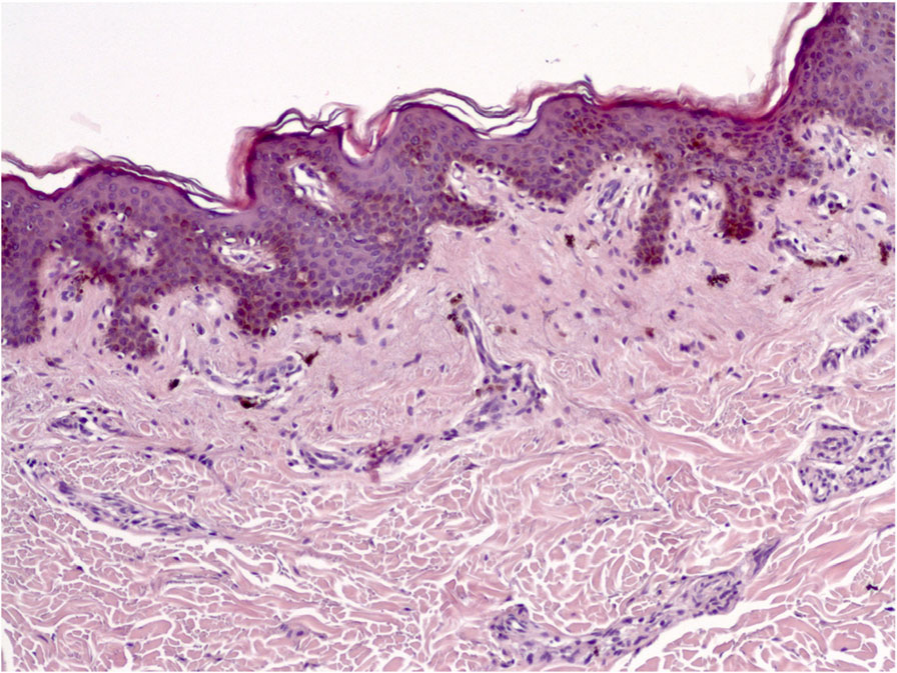
Hematoxylin and eosin stain 10x magnification of skin lesion biopsy showing focal hyperkeratosis, mild papillomatosis, and hyperpigmentation of the basal layer, consistent with the diagnosis of AN (see text for further details)

Excisional skin biopsy of the left areola lesion was performed and revealed focal hyperkeratosis, mild papillomatosis, and hyperpigmentation of the basal layer. Dermal papillae projected upwards as finger-like projections with prominent verticalisation of subepithelial vessels and scattered deposition of melanophages. The valleys between papillae showed mild acanthosis and seemed occasionally filled with keratotic material. These findings were overall consistent with a diagnosis of AN (Fig. 2). Commonly associated diagnoses as malignancies and endocrine disorders, as well as drug-related forms of AN, were excluded. Particularly, obesity and abnormalities in lipid profile as well as in glucose metabolism and insulin sensitivity were excluded. Indeed, patient’s body mass index was 24.3 kg/m^2^ and baseline fasting blood tests at admission – i.e. before starting ART – showed normal values of serum total cholesterol (3.62 mmol/L), low-density lipoprotein (LDL, 2.25 mmol/L), high-density lipoprotein (HDL, 0.91 mmol/L), triglycerides (1.01 mmol/L), as well as normal fasting glucose (5.1 mmol/L) and glycated hemoglobin (5.4%). Moreover, oral glucose tolerance test was performed as soon as AN diagnosis was confirmed and tested negative, too (fasting glucose: 4.8 mmol/L, 120-min glucose: 6.3 mmol/L). Fasting insulin at the time of oral glucose tolerance test was 7.2 μU/mL.

Interestingly, progressive improvement in AN size and pigmentation was observed after starting ART and complete regression was confirmed at one-year follow-up visit (Fig. 1b).

## Discussion and conclusion

AN is characterised by velvety, hyperpigmented plaques typically localised on intertriginous areas of the body surface. AN is commonly considered a cutaneous marker of insulin resistance. Indeed, the most frequently associated disorders are obesity, diabetes mellitus and every other condition linked to insulin resistance; however, in some cases, AN can be associated with internal malignancies, especially abdominal adenocarcinomas [1]. Genetic disorders and medications that cause hyperinsulinemia (i.e. glucocorticoids, HIV protease inhibitors, insulin, etc. [1, 4]) represent others secondary etiologies. Occasionally, AN occurs independently from all of the above-mentioned conditions.

In our case the patient did not suffer from any medical disorder usually linked to AN, however he was affected by HIV-1 infection. To our knowledge, this is the second case described of an AIDS subject diagnosed with *P. jirovecii* pneumonia and AN, the latter regressing once ART was initiated. Maltez et al. firstly reported in 1997 a case of a patient with AIDS complicated by three concurrent opportunistic infections and associated with AN [3]. Similarly to what observed in our case, AN disappeared after starting antiretroviral treatment.

The reasons behind this association are not completely clear, but this phenomenon might be linked to HIV-induced changes in the endocrine homeostasis. In fact, hyperinsulinemia seems to play a key role in the development of AN [5, 6], favoring the bond between insulin and growth factor receptors similar to insulin receptors, which stimulate keratinocytes and fibroblasts proliferation in the dermis [4]. According to literature [7, 8], HIV-1 patients have specific risk factors for metabolic syndrome and hyperinsulinemia compared with general population, such as chronic inflammation caused by persistent viral replication in the reservoirs despite effective antiretroviral treatments [9]. Moreover, other studies have described that some inflammatory mediators are involved in insulin resistance and diabetes development [10]. HIV-1 infection itself can contribute to the development of metabolic syndrome by decreasing the activity of lipoprotein lipase and hepatic lipase, allowing increase in triglycerides and total cholesterol blood concentrations [11, 12]. Indeed, it is also known that higher HIV-1 RNA levels are associated with lower LDL cholesterol and higher VLDL cholesterol and triglycerides [13].

Other data suggest that metabolism in HIV-1-infected patients is characterised by increased resting energy expenditure and a de novo hepatic lipogenesis [14]. Duro M et al. [7] reported that the risk of developing metabolic syndrome is lowered in HIV-1-infected patients when viral RNA load decreases after treatment. On the other hand, AN is also considered a manifestation of insulin resistance among patients with HIV-1 infection who are already on treatment with protease inhibitors [4, 15]. These findings are apparently contradictory, but suggest the presence of intricate mechanisms that may be involved in the occurrence of metabolic syndrome and AN among HIV-1 patients. Moreover, multiple endocrinopathies have been reported in HIV-1-infected subjects, such as dyslipidemia, osteoporosis and adrenal insufficiency [16]. In this setting, adrenalitis due to CMV infection is a well-recognised condition in AIDS patients [17], suggesting a possible role of CMV in the development of AN. In our case, CMV viremia was detected (1412 copies/mL), however no electrolyte disturbance neither hypotension suspicious for adrenal failure were observed.

In conclusion, our experience suggests that HIV-1 infection – particularly in the setting of AIDS – may be associated with AN, which in turn seems to respond successfully to ART. Based on this data, HIV-1 testing should always be considered in the differential diagnosis of AN.

## Data Availability

All data generated or analysed during this study are included in this published article. All original data is available in the corresponding author.

## Abbreviations

HIV-1: human immunodeficiency virus type 1
AN: acanthosis nigricans
AIDS: acquired immunodeficiency syndrome
CMV: cytomegalovirus
ART: antiretroviral therapy
VLDL: very low-density lipoprotein
LDL: low-density lipoprotein
HDL: high-density lipoprotein
